# Study protocol for a cluster-randomized split-plot design trial to assess the effectiveness of targeted active malaria case detection among high-risk populations in Southern Lao PDR (the AcME-Lao study)

**DOI:** 10.12688/gatesopenres.13088.1

**Published:** 2019-12-17

**Authors:** Andrew A. Lover, Emily Dantzer, Sophia Hocini, Ronaldo Estera, Francois Rerolle, Jennifer L. Smith, Jimee Hwang, Roly Gosling, Joshua Yukich, Bryan Greenhouse, Jerry Jacobson, Rattanaxay Phetsouvanh, Bouasy Hongvanthong, Adam Bennett

**Affiliations:** 1 Department of Biostatistics and Epidemiology; School of Public Health and Health Sciences, University of Massachusetts- Amherst, Amherst, Massachusetts, 01003-9304, USA; 2 Malaria Elimination Initiative, Global Health Group, University of California, San Francisco, San Francisco, CA, 94158, USA; 3 Department of Anesthesiology and Perioperative Medicine, David Geffen School of Medicine, University of California, Los Angeles, Los Angeles, CA, USA; 4 Health Poverty Action, Vientiane, Lao People's Democratic Republic; 5 Center for Global Health/ DPDM Malaria Branch/ US President’s Malaria Initiative, US Center for Disease Control & Prevention, Atlanta, GA, 30333, USA; 6 School of Public Health and Tropical Medicine, Tulane University, New Orleans, LA, 70118, USA; 7 Department of Medicine, Division of Infectious Diseases, University of California, San Francisco, San Francisco, CA, 94158, USA; 8 Independent Consultant, Los Angeles, CA, 90031, USA; 9 Department of Communicable Disease Control, Ministry of Health, Lao PDR, Vientiane, Lao People's Democratic Republic; 10 Center for Malariology, Parasitology and Entomology, Ministry of Health, Lao PDR, Vientiane, Lao People's Democratic Republic

**Keywords:** malaria, disease elimination, community-randomized study, high-risk populations

## Abstract

**Introduction: **Novel interventions are needed to accelerate malaria elimination, especially in areas where asymptomatic parasitemia is common, and where transmission generally occurs outside of village-based settings. Testing of community members linked to a person with clinical illness (reactive case detection, RACD) has not shown effectiveness in prior studies due to the limited sensitivity of current point-of-care tests. This study aims to assess the effectiveness of active case finding in village-based and forested-based settings using novel high-sensitivity rapid diagnostic tests in Lao People’s Democratic Republic (Lao PDR).

**Methods and analysis: **This study is a cluster-randomized split-plot design trial. The interventions include village-based mass test and treat (MTAT), focal test and treat in high-risk populations (FTAT), and the combination of these approaches, using high-sensitivity rapid diagnostic tests (HS-RDTs) to asses *P. falciparum* infection status. Within four districts in Champasak province, Lao PDR fourteen health center-catchment areas will be randomized to either FTAT or control; and within these HCCAs, 56 villages will be randomized to either MTAT or control. In intervention areas, FTAT will be conducted by community-based peer navigators on a routine basis, and three separate rounds of MTAT are planned. The primary study outcome will be PCR-based *Plasmodium falciparum* prevalence after one year of implementation. Secondary outcomes include malaria incidence; interventional coverage; operational feasibility and acceptability; and cost and cost- effectiveness.

**Ethics and dissemination: **Findings will be reported on clinicaltrials.gov, in peer-reviewed publications and through stakeholder meetings with Ministry of Health and community leaders in Lao PDR and throughout the Greater Mekong Subregion.

**Trial registration: **clinicaltrials.gov NCT03783299 (21/12/2018)

## Abbreviations

ACT: Artemisinin-based combination therapy; AE: Adverse event; AL: Artemether-lumefantrine; CRCT: Cluster randomized control trial; CMPE: Center for Malariology, Parasitology, Entomology (Lao PDR); DAMN: District anti-malaria nucleus; DBS: Dried blood spot; DHO: District health office; DSMB: Data safety monitoring board; FSAT: Focal screen and treat; FGD: Focus group discussion; G6PDd: Glucose-6-phosphate dehydrogenase deficiency; GMS: Greater Mekong Subregion; HC: Health center; HCCA: health center catchment area; HPA: Health Poverty Action; HRPs: High-risk populations (for malaria infection); HRP 2/3: ( *Plasmodium falciparum*) Histidine-rich protein 2/3; HS-RDT: Highly sensitive malaria rapid diagnostic test; HH: Household; IEC/BCC Information Education Communication / Behavioral Communication Change; IRS: Indoor residual spraying; KII: Key informant interview; Lao PDR: Lao People’s Democratic Republic; LIN: Long-lasting insecticidal nets; MSAT: Mass screen and treat; MTAT: Mass test and treat; MMPs: Mobile and migrant populations; PAMS: Provincial anti-malaria station; PN: Peer navigator; PQ: Primaquine; RDT: Rapid diagnostic test; PCR: Polymerase chain reaction; SAE: Serious adverse event; SLD-PQ: Single low-dose primaquine; VMW/VMV: Village malaria worker/volunteer; VHV: Village health volunteer; WHO World Health Organization.

## Introduction

While significant progress has been made in the last decade, malaria remains a major health issue globally, with an estimated 219 million cases in 2017 ^1^. The Greater Mekong Subregion (GMS, comprising Vietnam, Cambodia, Thailand, Myanmar, Lao PDR and China’s Yunnan Province) has achieved major progress towards the goal of malaria elimination by 2030, with a 74% reduction in cases, and a 91% decrease in reported mortality since 2012 ^2^. However, remaining caseloads generally occur in rural, marginalized populations throughout the region. These populations present important challenges for targeting of interventions by national malaria programs, and new interventions are needed to go this ‘last mile’ toward regional malaria elimination.

National malaria programs generally use passive data collection at health facilities to monitor disease burden. However, in elimination areas, additional approaches are required. The WHO recommends a shift to including active surveillance methods to identify clusters of asymptomatic parasite carriers. These active case detection activities can be triggered as a response to cases (reactive; or RACD); or may occur on a periodic basis in known higher burden areas (proactive; PACD). Moreover, proactive case detection can be conducted at the community scale (mass screen and treat (MSAT) or mass test and treat ( **MTAT**)) or in small geographic areas (focal screen and treat (FSAT) or focal test and treat ( **FTAT**)).

However, these approaches have not been effective in reducing transmission using standard RDTs, due to the limited sensitivity of these tests for low-level parasitemia ^3– 5^. In the GMS, few studies have measured the impact of active case detection, and all have failed to demonstrate impact on community-level prevalence using standard RDTs ^6, 7^. While PCR-based strategies have shown potential impacts in the region ^8^, this methodology is not field deployable, and case follow-up is challenging due to delays in data reporting.

While there has been limited documented impact on transmission of active case detection with standard RDTs, major investments in improved RDTs have led to the next generation of histidine-rich protein 2 (PfHRP-2) RDTs for *P. falciparum*, with an expected 8-10-fold greater sensitivity. The new highly sensitive Alere Malaria Ag P.f Ultra-Sensitive ^9^ (HS-RDT) became commercially available in 2017, with a primary intended use for active case detection in individuals with low parasitemia.

A second important challenge is targeting highest-risk populations (HRPs) for interventions. Active outreach is generally challenging due to the illegal nature of some activities, especially logging and hunting in protected forest areas; socio-cultural barriers in ethnic minorities; and limitations in human resources within the health sector. These challenges have been documented in the GMS at border screening posts ^10^, and in snowball sampling and related studies ^11, 12^.

One promising technique to address similar challenges in sampling hard-to-reach groups (especially in HIV/AIDS) is that of ‘peer-navigators’ (PNs) who are peer-group members who seek out persons like themselves and help them overcome modifiable barriers to health care ^13, 14^. These positions have several important differences from other outreach methods. Specifically, PNs need to have shared “lived experience” with the targeted groups including shared socioeconomic level, racial/ethnic identity, and common language(s), and the PN role must be formalized and include some level of training ^14^. Finally, their primary responsibility for malaria programming in Lao PDR is active case finding and treatment among targeted high-risk subpopulations.

To address these gaps, MTAT and FTAT, both utilizing HS-RDTs will be implemented and evaluated in an area of recent outbreaks in Champasak Province, Southern Lao PDR. The MTAT intervention will be conducted at the village level, targeting the entire community, while the FTAT intervention will be conducted in forest fringe areas, targeting forest-workers. Using a community-randomized split-plot design, this study will assess the effectiveness of both MTAT and FTAT independently and in combination with one another, in comparison to routine public sector activities in Champasak Lao PDR.

### Aims and objectives

The overall objective of this trial is to assess the effectiveness, feasibility, and acceptability of MTAT and FTAT in reducing *Plasmodium falciparum* infection prevalence.

The primary aim is to reduce the community-scale prevalence of *Plasmodium falciparum*. It is hypothesized that FTAT and MTAT when combined in a single interventional area will be associated with an overall 50% reduction of *P. falciparum* prevalence as measured by PCR. Secondary aims are a 50% reduction in reported incidence of *P. falciparum* in intervention areas relative to control areas; and to evaluate the feasibility, acceptability, and costings for these strategies.

### Study aim and objectives

In combination with novel strategies for accessing HRPs, we hypothesize that MTAT using the next generation of *Pf*HRP-2 RDTs can help bridge gaps in identification of high-risk asymptomatic individuals with low density parasitemia, allowing for targeting of this reservoir and thereby reducing transmission. Specifically, we hypothesize that in target areas, three rounds of MTAT using HS-RDTs in villages and FTAT in HRP sites will be more effective than no test and treat (standard of care) in reducing *P. falciparum* incidence and prevalence over a 12-month intervention period.

The primary aim is to evaluate the effectiveness of targeted test and treat activities using HS-RDTs compared to control for reducing the health center catchment- and village-level prevalence and incidence of *P. falciparum* within four districts in Champasak Province. Secondary objectives include determining risk factors for malaria infection; determining the cost-effectiveness, acceptability, and operational feasibility of MTAT and FTAT in this setting; assess the sensitivity and specificity of HS-RDTs relative to PCR; and to estimate the sizes of HRP populations in Champasak province.

## Methods and analysis

The SPIRIT guidelines for randomized trials ^15^ have been followed throughout the design and reporting of this study protocol; the completed checklist has been archived see (reporting guidelines ^16^).

### Study design

**Figure 1.**
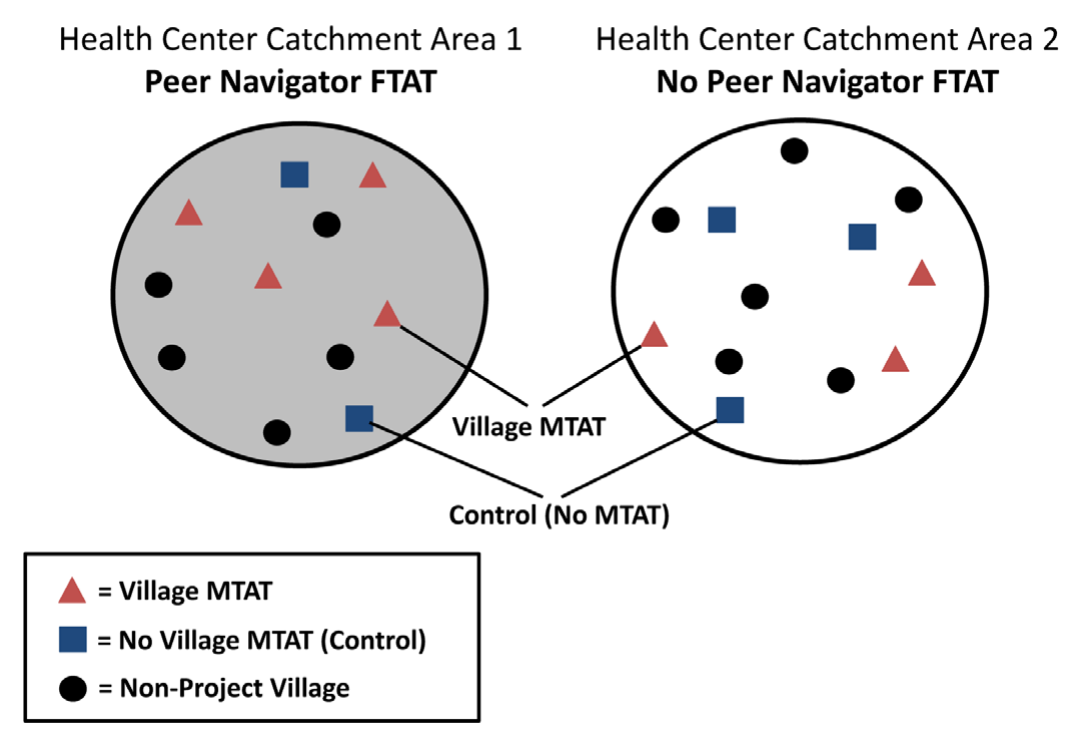
Schematic of split-plot study design, AcME-Lao trial.

This study will employ a cluster-randomized control trial (CRCT) and will randomize the interventions in two separate stages using a split-plot design ( Table 1 and Figure 1).

**Table 1.** Split-plot community randomized controlled trial design. AcME-Lao trial. (note: HCCA = health center catchment area; FTAT = focal test and treat; MTAT = mass test and treat; RDT = rapid diagnostic test; HS-RDT = high-sensitivity rapid diagnostic test).

Study arm	Treatment group	Intervention description	Number of HCCAs	Number of villages/village clusters per arm
**In health center catchments without FTAT (total of 7 HCCAs)**				
**1A**	Village-based test and treat	All members of target household population will be offered RDT- based testing with standard and HS-RDTs, with anyone testing positive for malaria treated as per national guidelines.	7	14
**1B**	Control	Standard of care		14
**In health center catchments with FTAT (total of 7 HCCAs)**				
**2A**	Village-based test and treat	All members of target household population will be offered RDT- based testing with standard and HS-RDTs, with anyone testing positive for malaria treated as per national guidelines. overlaid with FTAT.	7	14
**2B**	Control	Standard of care, overlaid on FTAT.		14

A split-plot design ^17– 19^ randomizes at two differing spatial scales, allowing one set of treatment/control (‘sub-plot’) areas to be nested within the second set of treatment/control areas (‘whole plot’). In this design, the first level, or ‘whole plot’ is often an intervention that is operationally difficult to conduct at a lower level. Randomization at two different spatial scales is necessary within this study, as PN-based FTAT will be operating in areas between villages, so randomization at the village level will not be feasible. Conversely, the MTAT campaigns will be organized at the village level to maximize the number of units available for randomization to ensure adequate study power. The split-plot design allows for greater power to detect differences in sub-plot effect sizes as well as the interaction between sub-plot and whole plot.

### Study setting and trial preparations

**Figure 2.**
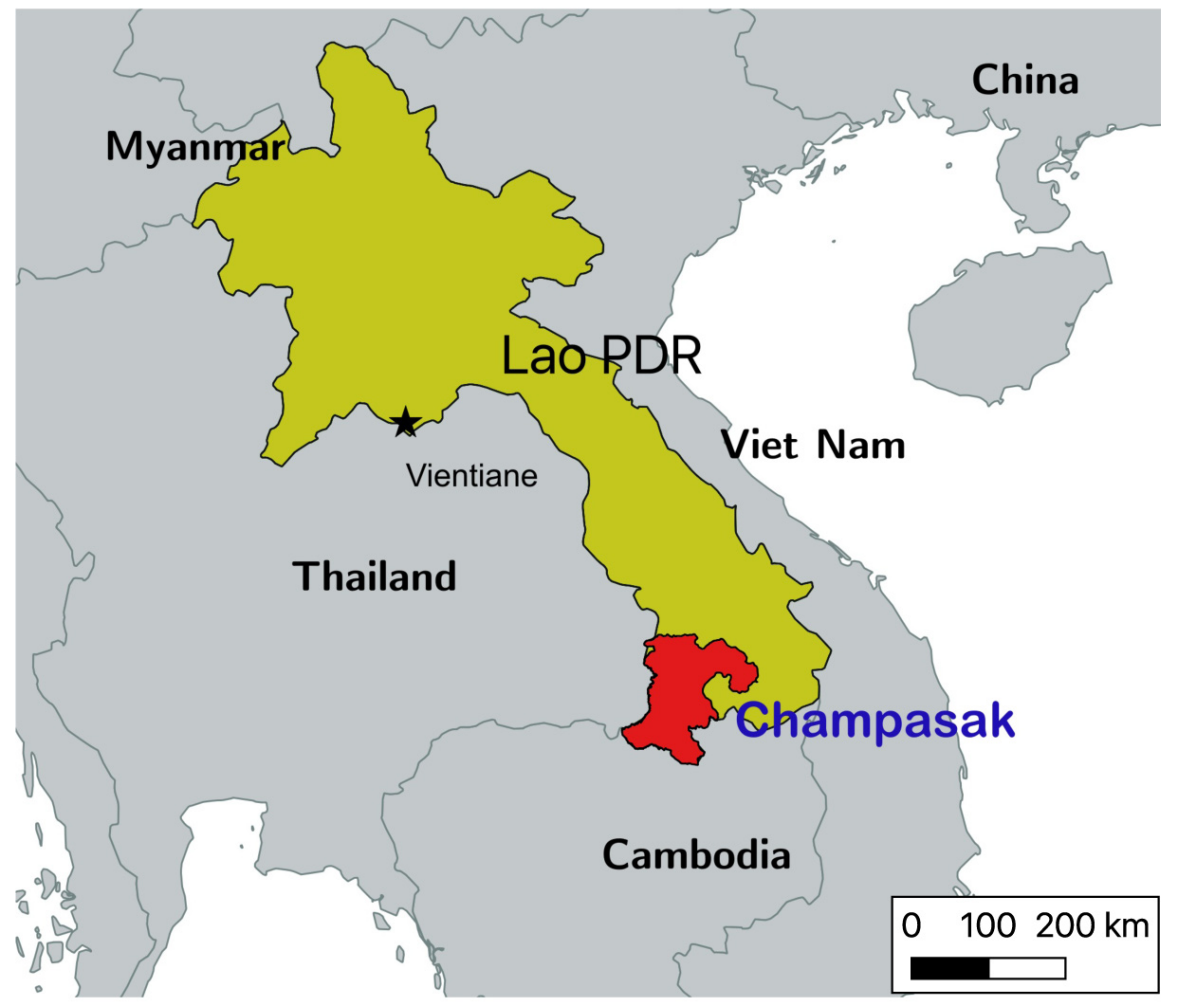
Location of Champasak Province, Lao People’s Democratic Republic.

**Figure 3.**
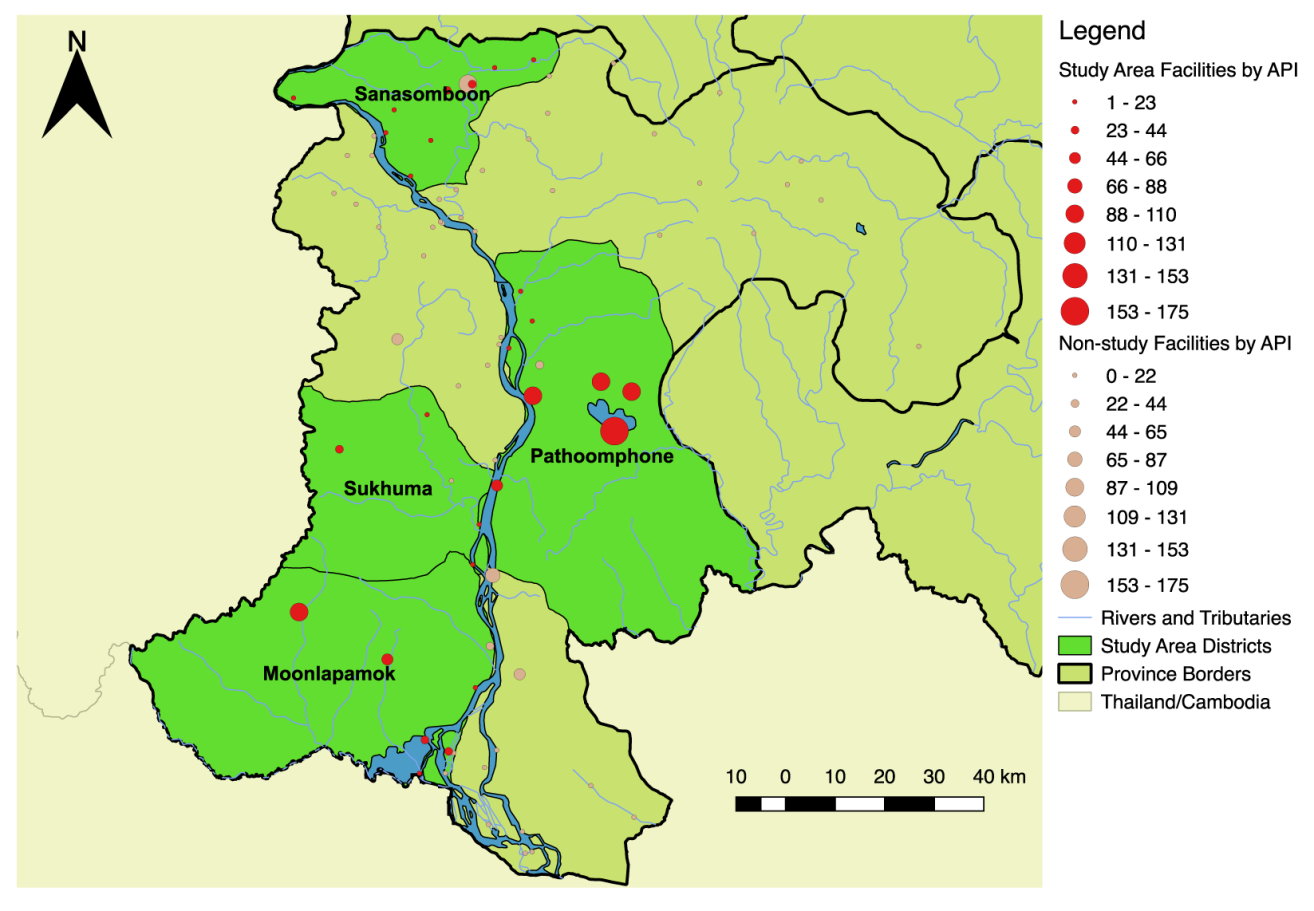
Overview of project districts, Champasak Province, Lao People’s Democratic Republic, showing all-species annual parasite indices (API) (2015).

This study will be carried out in selected health center catchments areas (HCCA) in four districts within Champasak Province (Mounlapamook, Panthampone, Sanamsaboun, and Soukhuma), targeting the highest-burden HCCAs ( Figure 2– Figure 3) which include those that were part of earlier unpublished formative studies conducted by our team in 2016–2017. The study area population, number of villages, total health facilities, and species-specific annual parasite indices (API) for 2015 can be found in Table 2.

**Table 2.** Study area population and confirmed case incidence, Champasak Province, Lao People’s Democratic Republic. (API = annual parasitemia index).

District	Total villages	Total population (2015 census)	Total health facilities	*Plasmodium* *falciparum* cases, 2015 (API)	*Plasmodium* *vivax* cases, 2015 (API)	Total cases 2015 (API)
Mounlapamook	50	38,800	8	944 (24.3)	1558 (40.2)	2593 (66.8)
Panthamphone	93	61,252	9	2529 (41.3)	2381 (38.9)	5082 (83.0)
Sanamsaboun	86	69,338	10	268 (3.9)	1564 (22.6)	1856 (26.8)
Soukhuma	62	58,445	5	639 (10.9)	788 (13.5)	1459 (25.0)

Currently, standard-of-care interventions in these districts include periodic mass distribution of ITNs, health facility level testing with RDTs, community case management by VMWs in high-burden areas, and IEC/BCC work through village malaria/health workers. There is currently no indoor residual spraying implementation, and case investigation only occurs during major outbreaks. The standard first-line treatment for confirmed uncomplicated *P. falciparum* malaria cases in Lao PDR is artemether-lumefantrine (AL); single low-dose primaquine (SLD-PQ) has recently been added to this regimen but is not widely available.

Prior to the trial, formative work had been occurring in this region since 2016. To prepare communities for intervention roll-out and survey data collection, community sensitization and engagement activities will be implemented. These include informational letters/requests to village authorities, malaria flyers and AcME study info brochure, posters advertising FTAT activities, and malaria IEC/BCC flipcharts for use in community sensitization meetings.

### Randomization

Study randomization will occur in two distinct stages.

***Randomization 1: Health center selection and randomization.*** A total of 14 HCCAs will be selected for inclusion based upon HCCA-level API and distance between HCCAs. HCCAs with higher API will be prioritized to improve power; where possible, directly neighboring HCCAs will not both be included to reduce contamination. Restricted randomization of the 14 HCCAs into either PN or control arms will be conducted, whereby HCCAs will first be matched into strata based upon HCCA-level API, population size, amount of forest cover, and non-contiguity of opposite arms. Restricted randomization in this way will ensure balance across intervention and control groups on potential confounding factors ^20^. Custom code, based upon *sample* and *randomizeR* in R software versions 3.4.1 ^21, 22^, will be used to implement all study randomizations.

***Randomization 2: Village selection and randomization.*** All villages within selected HCCAs will be mapped and restricted randomization conducted for 28 villages from among each of the two HCCA arms from randomization 1 (FTAT or control). Randomization 2 will randomize the 56 total study villages to either MTAT or control (no MTAT intervention). For each arm of randomization 1, suites of 28 villages that maximize distances between villages and/or include non-study buffer villages will be generated. From amongst these sets, a single set of 28 villages will be randomly selected for each arm of randomization. If possible, a public event will be held during which village elders randomize the units for full transparency of intervention allocation ^23^.

***Allocation concealment.*** After allocation, the specified interventions will be implemented in HCCAs and villages, but assignments will not be blinded to participants or investigators due to the nature of the interventions. However, all efforts will be made to ensure data collection teams will be blinded to these allocations for the end-line cross-sectional survey. For analysis of all primary outcomes the study assignment arm will be blinded by use of concealed codes within the final dataset.

### Study procedures

**Figure 4.**
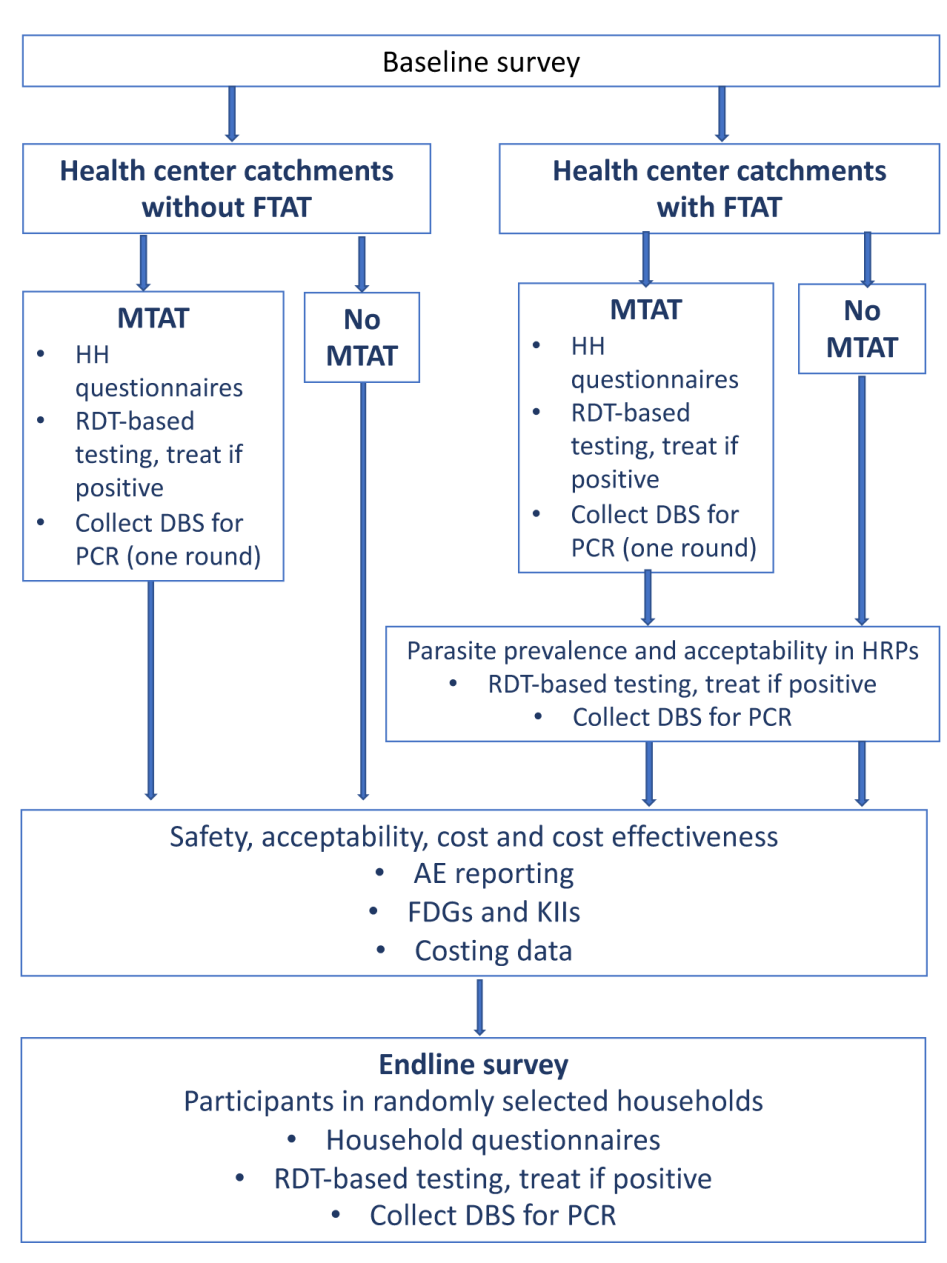
Flow of study procedures, AcME-Lao trial.

***General procedures.*** An overview of the study enrollment process and subsequent procedures is shown in Figure 4.

All survey instruments will be developed in English with input from local health staff. These will then be translated to Lao, and back-translated by a fluent bilingual health expert prior to field testing. Data will be collected via an ODK-based tablet application with internal range checks or paper-based with subsequent double-entry (see extended data ^24^), and will be stored on password-protected computers. Qualitative study components will use interview/discussion guides and a competency checklist (see extended data ^24^) will be developed using best practices in qualitative research ^25^. FGDs and KIIs will also be audio-recorded, transcribed, and the transcripts translated into English.

Within each of the 56 villages selected for study inclusion, survey staff will work with village authorities to update household ledgers, all participating baseline and MTAT households will be enumerated and GPS coordinates captured. These households will also be given a study ID card and corresponding barcode sticker (see extended data ^24^), which will be used throughout the study to identify repeat visits at each household, as well as for individuals to present at health facilities if they report for care.

***Baseline and endline surveys.*** At the baseline and end-line, cross-sectional surveys will be conducted to obtain an unbiased estimate of *P. falciparum* malaria parasite prevalence in each study arm. The baseline survey will be used to obtain baseline demographics, intervention coverage, and both standard and HS-RDT-based parasite prevalence to inform restricted randomization of study villages, and to assess the prevalence of any HRP2/3 deletions. The end-line survey will also test with both RDTs, but will be focused on the primary outcome for the overall study using PCR-based testing.

The survey questionnaire will capture household-level demographics, and assess potential risk factors for malaria infection, including forest-going and travel histories. All household members (residents and temporary visitors) aged 18 months and older will be invited to participate in an RDT and blood collection component. After informed consent, the study team will capture axillary temperature, and test each individual using both a standard and HS-RDT, and DBS collection. Survey and questionnaires in English and Lao are available as extended data ^24, 26^.

***Interventional eligibility.*** All household members aged >18 months will be invited to participate in MTAT activities, including RDT and HS-RDT testing. Treatment will be as per national guidelines with an age-appropriate course of artemether-lumefantrine (AL; in age-specific blister packages) and single low-dose primaquine (SLD-PQ) according to national treatment guidelines ^27^. The eligible study population for FTAT will include all persons aged 15 years and older approached at any non-village sites within the defined HCCAs who spent at least one night outside a formal village in the past one month, including individuals traveling into the HCCA who are willing and sufficiently able to communicate with PNs to assess their eligibility.

***Treatment of malaria cases.*** Treatment of any person with a positive RDT or HS-RDT during baseline surveys, MTAT or FTAT activities will be as per national guidelines with an age-appropriate course of AL (age-specific blister packages) and SLD-PQ according to national treatment guidelines ^27^.

Several groups will be ineligible for treatment and will be referred to local health facilities: all persons with severe disease; and any women who have a positive HS-RDT or RDT test and who i) suspect they are pregnant or 2) who are tested and positive with a human chorionic gonadotropin (hCG) rapid pregnancy test. Finally, measurement of lower PQ doses is challenging due to the physical size of 15-mg tablets, and therefore SLD-PQ will not be administered to children weighing less than 10 kg. MTAT teams will be provided with scales to weigh all eligible participants in order to determine the correct SLD-PQ treatment dose.

***Village-level mass test and treat with HS-RDTs (MTAT).*** The village-level test and treat intervention will be implemented in three rounds during the study period. The three rounds will be conducted with at least one-month separation between rounds, avoiding traditional rainy and harvesting seasons, as many individuals may not be available for household visits.

The sampling frame for all MTAT activities will consist of all permanent households within villages using updated village-level household registers. After advance notice of proposed activities to maximize participation, in selected villages, all households will be visited by MTAT teams comprised of local district, health center, and village malaria staff, with planned visitation of 100% of the households.

At each visit, a short demographic and malaria risk factor survey (see extended data ^24^) will be administered to the head of household, and all household members aged >18 months will be invited to participate in RDT and HS-RDT testing; DBS will also be collected from a randomized 25% subset of all households during one MTAT round.

***Focal test and treat (FTAT).*** In Lao PDR, some VMWs/VHVs, and many malaria post volunteers, also work in the forests, and fulfill criteria for working as peer navigators (PNs). Moreover, these staff have been trained by the national program to perform RDT-based testing. Efforts will be made to recruit PNs fluent in Lao, Khmer, local languages, and potentially Vietnamese, wherever possible. The main criteria for PN is being 18 or over, residing in and familiar with the HCCA, being literate and regularly travelling to forested areas.

The number of PNs will depend on total population, forest areas, and reported malaria burden. After being trained in data and sample collection, the primary role of PNs throughout the study will be to actively seek non-village based HRPs in forested areas, rice fields and plantations, and any other non-permanent settlements within target HCCAs, and conduct FTAT among all consenting individuals. Areas for potential recruitment by forest-based FTAT will be through three mechanisms: mapping of forested areas in each HCCA; travel to and snowball referral at all identified worksites/travel routes identified during formative studies; interviews after review of HC case reports for any forest-goer cases. Additionally, a subset of forest-goers will be invited to carry global positioning system (GPS) data loggers to capture their movement patterns in time and space.

***Acceptability and feasibility assessments.*** Multiple methods for assessing the feasibility and acceptability of MTAT and FTAT interventions as perceived by communities, HRPs, health sector staff, peer navigators, and study staff will be implemented over the course of the study. Focus group discussion (FGD) and key informant interview (KII) activities will be conducted at end-line and aim to reach theoretical saturation. PN supervisors will also directly observe PN activities, and will interview a subset of PNs to understand perceived FTAT challenges, facilitators, performance, and self-efficacy.

***Adverse event reporting.*** A passive case detection system including local public and private health facilities in the target areas, including referrals by VMWs/VHVs, will be used to collect AEs related to AL or SLD-PQ in the study. The completed forms will then be submitted to the data monitoring and safety board (DSMB) and study coordinators for review, classification as an adverse event or severe adverse event (AE or SAE respectively), and followed up as required. All participants receiving study drug will be provided with an informational sheet listing potential side effects and instruction to seek care at the nearest health center should they experience any of the defined symptoms or other adverse events. Community sensitization events and targeted IEC/BCC materials will also help increase community awareness of potential adverse events and encourage early care-seeking if events arise.

***Cost and cost-effectiveness.*** For the costing portion of the study, the cost per case investigation conducted; cost per additional positive case identified using MTAT in village-based populations and FTAT in forest-based HRPs; and the cost per case actively detected per person tested during test and treat will be assessed. The cost-effectiveness of test and treat will also be compared between village-based activities and peer-navigator led testing. Costing and analysis methods will follow principles of costing for health care programs ^28^ and analysis and presentation of results will follow the CHEERS guidelines ^29^. Costing will be conducted from the health-sector (provider) perspective and will utilize key informant interview and focus group data collection methods.

***Consent procedures.*** An informed consent form will be administered both verbally and in writing to all participants in the local language for participation in the cross-sectional surveys, MTAT interventions, and FTAT activities. Informed consent will be obtained from all study participants, including parental consent for any participant younger than 18 years of age. Participation is entirely voluntary for both the surveys and the intervention rounds. For children <6 years old, malaria testing will be based on consent from the parent or guardian. For children 6 to <18 years of age, the child’s oral assent will also be required.

***Data management and protection.*** Study teams and regular health staff will receive comprehensive training, as well as ongoing evaluation, supervision, and supplementary capacity building as necessary to ensure data quality and completeness.

Data collection team members will provide the contact information for study coordinators who can be contacted for any further information on the topics brought up in the interview, or for additional treatment if necessary. Data will be stored in a secure password-protected database; all paper forms will be stored in a locked location. All audio data files from KIIs and FDGs will be stored on encrypted hard drives accessible only to project staff, and will be retained only until transcripts are produced and verified. Prior to analysis, all data will be de-identified with the exception of geo-location codes, which are necessary for specific per-protocol analyses. A cleaned final dataset with full metadata will be deposited in an open-access repository (ucsf.edu; https://datadryad.org/; or OSF) as per funder requirements as soon as feasible pending completion of all analyses.

***Laboratory procedures.*** Parasitological testing will use standard RDT (CareStart Ag Pf/Pv, SD Bioline Cat #05FK80) and HS-RDT (SD Bioline Malaria Ag P.f High Sensitive Cat# 05FK140), followed by collection of four DBS on filter paper (Whatman Protein Saver 903, Cat# Z761575). All RDTs will be from quality-assured lots.

DBS cards will be left to air-dry before storage in plastic bags containing desiccant, and DBS will be stored at 4°C within one week, and at -20°C within one month; extracted DNA will also be stored at -20°C. DNA from DBS will be extracted using the Chelex method ^30^, and tested by RT-PCR with subsequent speciation for all samples found to be *Plasmodium*-positive.

Serology will be used to improve the identification of hotspots and estimate current and historical transmission intensities ^31^. Using DBS, ELISA assays will be performed using previously described methods; antigens will include merozoite surface protein-1 ( *Pf*MSP-1) and apical membrane antigen-1 (AMA-1) ^32^ and Pvmsp-1 and Pvcsp ^33^. Assays will be performed in duplicate and optical densities recorded with an ELISA reader, and/or other platforms including bead array or protein microarrays (e.g., multiplex antibody detection assay as previously described). ^34^

All molecular and serological analyses will be for research purposes only and will not inform patient care.

### Outcomes and measures

The primary outcome measure will be PCR-based *Plasmodium falciparum* prevalence in study villages as measured during the endline survey in all persons > 18 months old. This will be complemented with the HS-RDT parasite positivity rate during the same survey.

The coverage of MTAT activities will be measured in two ways. Operational program coverage will be defined as the proportion of individuals ≥18 months old and households visited and offered the MTAT interventions within the target areas. The effective program coverage is defined as the proportion of individuals (≥18 months old) that agreed to participate in the MTAT intervention from all eligible individuals.

The community-level confirmed *P. falciparum* malaria parasite incidence will be defined as the number of outpatient department malaria confirmed and suspected cases per person per year for each village, as ascertained from the health facility registers, utilizing village population size estimates for the exposure denominator.

A more detailed description of outcomes and metrics can be found in Table 3.

**Table 3.** Outcomes and indicators. AcME-Lao split-plot community-randomized trial, Lao People’s Democratic Republic. (note: HCCA = health center catchment area; FTAT = focal test and treat; MTAT = mass test and treat; RDT = rapid diagnostic test; HS-RDT = high-sensitivity rapid diagnostic test).

Outcome	Measurement metric
*Plasmodium falciparum* prevalence in all persons aged ≥18 months	Prevalence of infection detected by PCR
*P. falciparum* malaria case incidence among all ages, matched to village by name	Cumulative incidence by RDT by routine testing at reporting sites in districts (health centers, PPM sites, and district hospitals)
HS-RDT and RDT test positivity rate	HS-RDT and RDT positivity rate data from village based MTAT-HS
Total and confirmed outpatient (OPD) *P. falciparum* malaria case incidence among all ages at the health facility level	Cumulative incidence by RDT by routine testing at reporting sites in districts (health centers, PPM sites, and district hospitals)
HS-RDT and RDT test positivity rate	HS-RDT and RDT positivity data from FTAT in forest/rice-field based HRPs
Cost and cost-effectiveness of interventions.	Cost using standard procedures for costing methods and cost-effectiveness ^28^ and will be based on a health-sector (provider) perspective.
*P. falciparum* prevalence by each testing methodology	Prevalence of infection detected by PCR, RDT, and HS-RDT
*P. vivax* prevalence in all persons aged ≥18 months	Prevalence of infection detected by PCR
Total and confirmed outpatient (OPD) *P. vivax* malaria case incidence among all ages, matched to village by name	Cumulative incidence by RDT by routine testing at reporting sites in districts (health centers, PPM sites, and district hospitals)
Acceptability and feasibility of MTAT	Proportion of survey respondents who strongly disagree, disagree, are ambivalent, agree and strongly agree on the importance and acceptability of community-based MTAT
Acceptability of FTAT	Proportion of HRP survey respondents who strongly disagree, disagree, are ambivalent, agree and strongly agree on the importance and acceptability of peer navigator-based FTAT
Feasibility of FTAT	Proportion of peer navigators who rate conducting the FTAT intervention as very easy, somewhat easy, somewhat difficult, and very difficult, and changes in proportions over time

### Sample size and power calculations

***Community-level malaria parasite prevalence.*** Sample size for the primary evaluation at the end-line survey was determined based on the power to detect a difference between MTAT and control, as well as between MTAT combined with FTAT and control, assuming a 30% reduction in prevalence in PN areas compared to control. A simulation-based approach was performed using R software ^21^ version 3.4.1 and the - *sim.glmm*- package ^35^.

With an assumption of 4% *P. falciparum* prevalence by PCR and a between facility and village variance of 0.10, in order to detect a 50% difference in the prevalence of *P. falciparum* between MTAT and control, with 80% power at a 5% significance level, a total of 14 village clusters per arm (56 villages across all arms) with 150 persons sampled per cluster (8,400 persons, or 1,680 households). To account for potential non-response rates of 10% at the HH-level, a total of 1,848 households will be sampled. These power calculations assumed a realistic 30% reduction in prevalence based upon the PN intervention independently; while the trial will not have adequate power to detect this level of difference, a reduction of 50% is detectable.

The sample size for the baseline survey (1,232 households and 6,160 persons, with 10% non-response) will be sampled to give a *P. falciparum* prevalence with precision of 4.0% (95% CI: 3.3-4.7%; binomial exact) in each arm.

***Community-level confirmed P. falciparum malaria parasite incidence.*** The confirmed parasite incidence from all reporting units (including HCs, district hospitals, VMWs with RDTs) will be captured throughout the study with support from study staff. In the months prior to the start of study activities, all villages will be mapped, and trainings conducted with health facility staff to systematize the collection of village names at health facilities for confirmed malaria cases. Routine supervision of health facility staff will be conducted to ensure accurate recording of village names in health facility registers throughout the trial.

Using simulation-based sample size calculations with Poisson-distributed annual case counts per village, an expected baseline *P. falciparum*-specific API of 6 per 1,000, with 14 HCCAs, a between village coefficient of variation of 0.004, a between facility variation of 0.003 (Lao stratification database, variance of API across all included HCCA = 0.0039), and an average village population size of 750 (750 person-years of follow-up), the study will be adequately powered at 0.90 to detect a 50% reduction in *P. falciparum* incidence for the test and treat intervention, and at 0.80 to detect a combined 50% reduction due to village-based test and treat, and a 30% reduction due to PN test and treat.

### Statistical analysis

 All analyses will be performed using R software (version 3.6, or current versions at time of analysis), and/or Stata (version 15 or 16; StataCorp, College Station Texas US).

***Primary outcomes.*** The effectiveness of the interventions will be assessed as *P. falciparum* prevalence via PCR at end-line using generalized linear mixed effects models with separate random intercepts to allow for clustering within villages and health center catchments. The binomial distribution will be used to analyze prevalence outcomes (logistic regression). All main analyses will be analyzed as intention-to-treat, and all survey clusters will be analyzed within the intervention group assigned at randomization, regardless of adherence. Primary effect measures will be using fixed effects for village MTAT intervention, PN FTAT intervention, and an interaction term for the combined interventions.

Secondary analyses will include adjustment for age, sex, health center-catchment and village-level baseline prevalence of *P. falciparum* parasitemia by PCR, and other potential confounders, and a per-protocol analysis of the primary effect estimate. RDT-based prevalences will be compared using a χ ^2^ test, as well as logistic regression models to account for potential confounding factors.

Population-based coverage of MTAT interventions will be estimated at individual and household level as the percent of the population that received a visit from the intervention teams to offer the MTAT interventions, among those eligible for inclusion. This will be obtained from a combination of MTAT program data and enumeration data. Additionally, the proportion of individuals accepting the MTAT interventions ≥3 months, among those eligible for inclusion in the intervention, will be estimated, providing an estimate of the effect coverage of each program using household enumeration data and remote sensing data. Where possible individual, household and community level factors associated with coverage will be assessed using mixed effects logistic regression.

Analysis of cumulative malaria incidence will be on an intention-to-treat basis using monthly counts of confirmed malaria cases from the health facility registers. Time-series Poisson or negative binomial model with random intercepts at the health center catchment and village levels. These analyses will include a fixed effect for each study arm, an interaction term for the combination of MTAT and FTAT, and a fixed effect for time period (pre- and post-intervention). The interaction between these two terms will be the primary effect measure (also known as the difference-in-differences estimator).

### Study timeline

The implementation period was November 2017- November 2018.

## Ethics and dissemination

### Ethical approvals

Ethical approval in Lao PDR has been obtained from the Ministry of Health review board (reference number 2017-075), and from the UCSF IRB (reference number 17-22577). The trial is registered at clinicaltrials.gov ( NCT03783299) on 21 December 2018. And additional approval for UMass was obtained through a reliance agreement with UCSF (dated 12/18/2018).

The trial was retrospectively registered due to clerical errors discovered during the study which required re-registration.

### Monitoring and auditing

***DSMB.*** The project will follow US National Institutes of Health (NIH) guidelines for establishing a data safety monitoring board (DSMB). The DSMB will be established prior to any data collection as part of this study. The primary responsibilities of the DSMB will be to periodically review and evaluate the accumulated study data for participant safety, study conduct and progress, and, when appropriate, efficacy, and 2) make recommendations to investigators concerning the continuation, modification, or termination of the trial. Membership of the DSMB will consist of five independent experts in malaria control, diagnosis, case management and epidemiology, and one member of the research team to advise and clarify study activities for the independent experts.

No data on futility or benefit of the intervention will be estimable during the course of the trial as the timing of outcome data collection precludes developing stopping rules based on outcome data collected during implementation. Safety concerns associated with the wide-scale use of AL + SLD-PQ, although unexpected, will form the basis of development of a stopping rule. The stopping rules for this trial will be based on detection of a significantly higher rate of mortality, hospitalization for possible drug-related events, or any other SAE in the MTAT villages versus actual rates in the control (non-MTAT) villages. Further, any investigated SAE that results in death that is found due to the administration of AL + SLD-PQ will be grounds for stoppage.

### Dissemination

The finalized protocol has been filed at http://www.clinicaltrials.gov/, and conforms to CONSORT recommendations for cluster randomized trials ^36^.

A final dataset with full metadata will be deposited in an open-access repository (ucsf.edu; https://datadryad.org/; or similar) as per funder requirements as soon as feasible pending completion of all analyses.

### Study status

This trial was initially registered at clinicaltrials.gov ID number NCT03783299, on December 21 2018. This manuscript is based upon protocol version 4.0 (November 2017); data collection was completed on December 31, 2018. Analysis of clinical samples is currently on-going.

## Discussion

This community-randomized trial is designed to rigorously evaluate the impact of HS-RDTs through two parallel and complementary active case detection strategies. The fidelity of the implementation will be measured by the population-level coverage of MTAT, and the geographic scope of FTAT.

There are several potential challenges to implementation, including changes in forest-going behaviors due to environmental or political pressures; a decrease in malaria cases, potentially compromising power; or a large increase in malaria or other infectious disease (i.e., dengue), which could overwhelm local health capacities. Other potential limitations include the presence of *P. falciparum* HRP2/3 deletions in study areas ^37^, where the impact of HRP2-based test and treat could be severely compromised. Other threats include the dynamic nature of HRPs, who may freely transit between intervention and non-intervention arms, or if a large influx occurs could overwhelm FTAT activities. Finally, this study will occur in the midst of routine public sector, and partner activities.

Lao PDR has made important progress towards malaria elimination in recent years through wide-scale deployment of standard interventions. The last cases however, maybe require more refined and targeted efforts in remote and forested areas. Moreover, these interventions must be scale-able, and cost-effective in light of inherently limited budgets. Data and results from this study will be used to develop evidence-based strategies to inform next steps towards malaria elimination in Lao PDR and throughout the GMS.

## Data availability

### Underlying data

No data are associated with this article

### Extended data

Open Science Framework: AcME-Lao Trial Extended Data. https://doi.org/10.17605/OSF.IO/CY5GB ^24^

This project contains the following extended data:

- F01_Cross-sectional Household Survey Informed Consent_en_clean.docx (Cross-sectional Household Survey Informed Consent, English)
- F02_Baseline Survey Questionnaire_en_clean.docx (Baseline/End-line Household Survey Questionnaire, English)
- F03_Individual Blood Collection Informed Consent_en_clean.docx (Individual Blood Collection Informed Consent, English)
- F06_Village MTAT Monitoring Survey_en_clean.docx (Village MTAT Monitoring Questionnaire, English)
- F07_MTAT RDT & Blood Collection Informed Consent_en_clean.docx (MTAT Individual Blood Collection Informed Consent, English)
- F09_PN_FTAT_Survey_en_clean.docx (Focal Test and Treat (FTAT) Survey, English)
- F14 FGD and KII Informed Consent_en_clean.docx (AcME Lao FGD and KII Informed Consent Form, English)
- F15 MTAT Team Focus Group Guide_en_clean.docx (MTAT Team Focus Group Discussion Guide. English)
- F26 HRP In-Depth Interview Form_en_clean.docx (AcME Lao Study High-Risk Population (HRP) Member Interview Guide, English)
- ID_sticker_AcME_Lao_HH.pdf (Study ID card)
- ID_sticker_AcME Lao FTAT.pdf (Barcode sticker)

Open Science Framework: AcME-Lao Trial Extended Data- Lao versions. https://doi.org/10.17605/OSF.IO/6A3ZY ^26^

This project contains the following extended data:

- F01_Cross-sectional-Household Survey_IC_Lao_clean.docx (Cross-sectional Household Survey Informed Consent, Lao)
- F02_Baseline Survey Questionnaire_Lao_clean.docx (Baseline/End-line Household Survey Questionnaire, Lao)
- F03_Individual blood collection_IC_Lao_clean.docx (Individual Blood Collection Informed Consent, Lao)
- F06_Village MTAT Monitoring Survey_Lao_clean.docx (Village MTAT Monitoring Questionnaire, Lao)
- F07__MTAT_RDT_BloodCollection_IC_Lao_clean.doc (MTAT Individual Blood Collection Informed Consent, Lao)
- F09_PN_FTAT_Survey_Lao_clean.docx (Focal Test and Treat (FTAT) Survey, Lao)
- F14_FGD_KII_IC_Lao_clean.doc (AcME Lao FGD and KII Informed Consent Form, Lao)
- F15_MTAT_Team FGD Guide_Lao_clean.docx (MTAT Team Focus Group Discussion Guide, Lao)
- F26 HRP In-Depth Interview Form_Lao_Clean.docx (AcME Lao Study High-Risk Population (HRP) Member Interview Guide, Lao)

### Reporting guidelines

SPIRIT checklist for “Study protocol for a cluster-randomized split-plot design trial to assess the effectiveness of targeted active malaria case detection among high-risk populations in Southern Lao PDR (the AcME-Lao study)”. https://doi.org/10.17605/OSF.IO/U4CJ5 ^16^

Data are available under the terms of the Creative Commons Zero "No rights reserved" data waiver (CC0 1.0 Public domain dedication).
