# Supplementary material for: Study protocol for a cluster-randomized split-plot design trial to assess the effectiveness of targeted active malaria case detection among high-risk populations in Southern Lao PDR (the AcME-Lao study)
Source: Gates Open Res. 2019 Dec 17;3:1730. [Version 1] doi: 10.12688/gatesopenres.13088.1 (PMC7019195; doi:10.12688/gatesopenres.13088.1)
Supplement: Peer review history

# Peer review: Reviewer response for version 1

**DOI:** 10.21956/gatesopenres.14234.r28469

**Published:** 2020-03-02

**Authors:** Menno R. Smit

**Affiliations:** 1 Liverpool School of Tropical Medicine, Liverpool, UK; 2 Amsterdam University Medical Center, Amsterdam, The Netherlands

Lover *et al.*’s manuscript describes the study protocol for a large cluster randomised trial on the effectiveness of targeted active malaria case detection in Lao that ran from November 28, 2017 until December 31, 2018. The manuscript summarizing the protocol is well written. The study design is clearly described and well suited to address the research question. The protocol was previously approved by participating IRB’s and the trial was registered with clinicaltrials.gov retrospectively “due to clerical errors” on December 21,2018. The manuscript’s appendices include the full amended protocol version 4 dated November 5, 2017 and a completed SPIRIT checklist.

1. The study protocol states that it was amended. It may be useful to state in the manuscript whether any amendments to the study protocol were made after study start, and, if so, what the amendments were. If no amendments were made after the study start, this could also be stated.
2. “The trial was retrospectively registered due to clerical errors discovered during the study which required re-registration.” To support this statement it may be useful to include the original trial registration number, or to give further explanation why this cannot be provided. If pre-trial registration was an oversight, this could also simply be stated “The trial was retrospectively registered due to earlier oversight.” Now readers may be left wondering what exactly happened.
3. “Abstract, Introduction” and “Introduction, 3 ^rd^ paragraph”: Suggest to insert ahead of “due to” one of the following options: “in part”, “partially”, “likely”, or “largely” to recognize other potential factors contributing to the lack of effect of RACD in previous studies.
4. “Aims and objectives”: The use of the words “aim” and “objective” have likely been interchanged. It would be helpful to start with an overall aim, followed by a list of specific primary and secondary objectives/outcomes.
5. “Study aims and objectives”: There seem to be two similar sections, perhaps for the sake of clarity they can be merged?
6. “Allocation concealment”: “For analysis of all primary outcomes the study assignment arm will be blinded by use of concealed codes within the final data set.” How many primary outcomes are there? From the manuscript up until this point I had the impression it was only “prevalence by HS-RDT”, however in appendix protocol I see there are 4?
7. “Village-level mass test and treat with HS-RDTs (MTAT)”: MTAT will avoid the rainy season as residents may not be available, however from a transmission perspective it may be the most impactful time to intervene. Was any population-level modelling performed regarding the timing of the intervention?
8. “Outcomes and measures”: It may be helpful to move this section forward in the manuscript, see for example the order of components in the SPIRIT checklist. E.g. it would be good to know the outcomes before going into the data collection methods, etc. Furthermore, as the 4 outcomes listed in this section are deemed primary outcomes in the protocol, it would be good to indicate as such here.
9. “Table 3”: Indicate which are primary outcomes.

Congratulations to the authors on a thoughtfully designed study and well written manuscript.

I confirm that I have read this submission and believe that I have an appropriate level of expertise to confirm that it is of an acceptable scientific standard.

# Peer review: Reviewer response for version 1

**DOI:** 10.21956/gatesopenres.14234.r28483

**Published:** 2020-02-17

**Authors:** Kamala Thriemer

**Affiliations:** 1 Global and Tropical Health Division, Menzies School of Health Research, Charles Darwin University, Darwin, NT, Australia

This is a well written study protocol aiming to assess the effectiveness of MTAT and FTAT using high-sensitivity RDTs. Since the study has already been completed my comments mainly refer to potential clarifications.

1. The terms “PN based FTAT” and “FTAT” or just “PN” are used interchangeably. I suggest streamlining this across the protocol for clarity.
2. Section “general procedures”: the authors state that individuals in the MTAT households will receive ID cards for identification during household visits and for when they present at health facilities for care. It is unclear for which outcome measure this data will be used.
3. The section on FTAT implementation is fairly vague and it is not clear how quality and coverage will be ensured. How can the authors ensure that if no difference between FTAT and controls is found, this is a true finding rather than very low coverage in the FTAT clusters?
4. In the section on outcome measures the authors state that MATA coverage is measured as the proportion of individuals that agreed to participant. It might be useful to break this down depending on how many rounds individuals participated in (only using the first round might artificially increase the coverage).
5. In the same section the authors state that community level *P. falciparum* incidence will be calculated using population size estimates as denominator and cases presenting at the health facility as numerator. How sure are we about those estimates? Is there data on health seeking behavior that would suggest the majority of cases would be detected?

I confirm that I have read this submission and believe that I have an appropriate level of expertise to confirm that it is of an acceptable scientific standard.

# Peer review: Reviewer response for version 1

**DOI:** 10.21956/gatesopenres.14234.r28482

**Published:** 2020-02-12

**Authors:** Lorenz von Seidlein

**Affiliations:** 1 Centre for Tropical Medicine and Global Health, Nuffield Department of Medicine, University of Oxford, Oxford, UK

Lover *et al.* have drafted the protocol for a large cluster randomised trial for publication. The study design is sophisticated and well suited to address the research question. The manuscript is well written. The implementation period for this trial was from November 2017 and November 2018. It would be reasonable to read the findings in a peer reviewed journal at this stage. The protocol could be of historical interest for readers trying to understand aspects of the trial or researchers looking for inspiration in how to write a protocol. I believe the manuscript serves well for both purposes. In retrospect it may be difficult to understand what made the investigators think they their screen and treat approach would do better than earlier attempts. The authors provide a useful explanation *“… next generation of histidine-rich protein 2 (PfHRP-2) RDTs for P. falciparum, with an expected 8-10-fold greater sensitivity.”* We know now that this expectation was way off, but it is important to document that the study was driven by the expectation that these HS RDTs can boost sensitivity.

**Minor suggestions:**

**Intro:**

-  “hunting in protected forest areas;” isn’t that the same as poaching?
- “It is hypothesized that FTAT and MTAT when combined in a single interventional area will be associated with an overall 50% reduction of P. falciparum prevalence as measured by PCR.” Why 50%? It looks a bit arbitrary – a brief justification could be helpful for the reader?

**Methods:**

- Split plot design = is this a variation of a factorial design? If so, it may help the reader to explain differences and communalities with a factorial design? I suspect readers are more familiar with the term factorial study designs than split plot designs?
- “all participating baseline and MTAT households will be enumerated and GPS coordinates captured” what is a baseline household?

**Funding:**

- I didn’t find the funding source for this trial. The name of the publication “Gates …” may provide a hint where the money is coming from, but it would be could to have that spelled out?

I confirm that I have read this submission and believe that I have an appropriate level of expertise to confirm that it is of an acceptable scientific standard.

# Peer review: Reviewer response for version 1

**DOI:** 10.21956/gatesopenres.14234.r28382

**Published:** 2020-01-27

**Authors:** Daniel M. Parker

**Affiliations:** 1 Department of Population Health and Disease Prevention, University of California, Irvine (UC Irvine), Irvine, CA, USA

Overall this is a well-written and well-thought-out protocol. As it appears to have already been funded and implemented, my comments here related more to the background text and impetus of this work. Without seeing how the data are analyzed, presented, and generalized, it is difficult to criticize the methods (which largely seem reasonable to me).

 In abstract – I suggest changing “asses” to “assess” in the Methods and analysis section

 Page 3 column 1 paragraph 4: “However, these approaches have not been effective in reducing transmission using standard RDTs, due to the limited sensitivity of these tests for low-level parasitemia.”

 This is one likely reason that these approaches have not been effective. There are other likely reasons as well. All of the approaches discussed here (RACD, PACD, MSAT, MTAT, FCAT) have a geographic focus, even if that focus differs (from households, to groups of households, to villages, etc) If the goal of these approaches is to disrupt transmission (rather than to simply detect extra cases) then there is an inherent assumption that the spatial unit that is being targeted is on the scale at which transmission occurs. For example if an entire village has a high proportion of low parasite density, asymptomatic and infectious individuals, but only a small portion of that village (say a cluster of houses around a symptomatic infection) is screened and treated or presumptively treated; it is unlikely to significantly disrupt transmission. The same is likely to be true at larger scales. If clusters of villages are highly connected and all have high prevalence of asymptomatic infectious individuals, targeting an entire village is unlikely to be effective at disrupting transmission (at least over time).

 Another reason that these approaches may not have been effective has to do with the relative importance of low-density infections in overall transmission. Arguably, high density infections are more infectious than low-density infections for any given unit of time. A symptomatic individual with high parasitemia, who waits for a few days before being diagnosed and treated, may be more important than several asymptomatic low parasitemia individuals. Delayed treatment seeking behavior can frustrate efforts at disrupting transmission and may not be detected by any of the methods discussed here (unless the individual is within the spatial unit that is being screened or pre-emptively treated).

 All of these approaches (and others not listed, such as MDA) have significant labor and financial costs that may not be practical in places where the malaria burden is heaviest or most persistent.

 I’m not suggesting that the limited sensitivity of RDTs is unimportant – but I would argue that it is not the only important factor here. Even if the diagnostic used was highly sensitive it is not clear to me that these approaches alone will be capable of reducing transmission.

 Page 3 column 2 paragraph 1: “While PCR-based strategies have shown potential impacts in the region, this methodology is not field deployable, and case follow-up is challenging due to delays in data reporting.”

 Can you please specify what is meant by “field deployable”? There have definitely been PCR-based interventions for malaria in the region, even in difficult field settings.

 Page 3 column 2 final paragraph: “The primary aim is to reduce the community-scale prevalence of *Plasmodium falciparum*.” Can you please define “community” here? Is it a single village? Could a community be larger than a single village (related to my comments about spatial scale)?

 Page 6 column 1: Can you be a bit more specific about how the locations were selected? Were they health care catchment areas with the highest API in 2015?

 Page 9 column 2: When will the follow up prevalence surveys be conducted? Is there sufficient time between the intervention and the follow-up survey to say whether or not transmission has effectively been disrupted, given that this region generally has unstable and highly seasonal transmission? Presumably this work has already been done, but I would suggest that > 12 months follow-up be considered.

 More generally:

 Why not mass drug administration, which could also be triggered by epidemiological surveillance?

 The target of the FTAT component (people/populations living outside the village) is a complicating factor. I’m sure the authors have considered this, but transient and mobile populations aren’t all the same in demography, mobility, or immunity. Even if there are positive results from this study, it may be difficult to make broad generalizations. Surely detecting cases in such populations is good practice when possible, and will result in better health of these individuals, but the overall impact of on larger scale population health must be influenced by many related factors – including how remote these populations are, how large they are, how infectious infected individuals are and how quickly they recieve diagnosis and treatment (even in between FTAT), how frequently they move, etc.

I confirm that I have read this submission and believe that I have an appropriate level of expertise to confirm that it is of an acceptable scientific standard.
